# Internet-based indicated prevention of anxiety and depression disorder onset three-arm randomized clinical trial

**DOI:** 10.1038/s41746-025-01990-8

**Published:** 2025-10-01

**Authors:** Anna-Carlotta Zarski, Kiona K. Weisel, Thomas Berger, Tobias Krieger, Michael P. Schaub, Matthias Berking, Dennis Görlich, Corinna Jacobi, Rosa Baños, Cristina Botella, Rocio Herrero, David D. Ebert

**Affiliations:** 1 Division of eHealth in Clinical Psychology, Department of Clinical Psychology, Philipps University of Marburg, Marburg, Germany; 2 Department of Clinical Psychology and Psychotherapy, Friedrich-Alexander-University Erlangen-Nürnberg, Nuremberg, Germany; 3 Department of Clinical Psychology and Psychotherapy, University of Bern, Bern, Switzerland; 4 Swiss Research Institute for Public Health and Addiction (ISGF), associated to the University of Zurich, Zurich, Switzerland; 5 Institute of Biostatistics and Clinical Research, University of Münster, Münster, Germany; 6 Institute for Clinical Psychology and Psychotherapy, Technical University of Dresden, Dresden, Germany; 7 University of Valencia, Valencia, Spain; 8 CIBER Pathophysiology of Obesity and Nutrition (CB06/03), Carlos III Institute of Health, Madrid, Spain; 9 University Jaume I, Castellon, Spain; 10 University of Zaragoza, Teruel, Spain; 11 Chair for Psychology & Digital Mental Health Care, Technical University of Munich, Munich, Germany

**Keywords:** Psychology, Health care

## Abstract

Preventing mental disorders is important to avoiding clinical conditions. This study evaluated the efficacy of internet-based indicated prevention for anxiety and depressive disorders. In a three-arm randomized controlled trial, 566 adults with subthreshold anxiety (GAD-7 ≥ 5) and/or depressive symptoms (CES-D ≥ 16), but no clinical diagnosis in the past six months (MINI 6.0), were assigned to either an individually (IG-IMI, *n* = 186) or automatically (AG-IMI, *n* = 189) guided digital intervention, or waitlist control (WLC, *n* = 191). The digital intervention comprised 8 transdiagnostic, self-tailored, CBT-based sessions. The primary outcome was time to onset of any anxiety or depressive disorder over 12 months, assessed via blinded diagnostic interviews (MINI). AD/DD onset was 19.4% in IG-IMI, 14.8% in AG-IMI, and 30.9% in WLC. Cumulative incidence was 23.1% (IG-IMI), 20.7% (AG-IMI), and 36.0% (WLC; *p* < 0.001). Hazard ratios were 0.59 and 0.47; NNTs were 7.76 and 5.79. Both individually guided and automated interventions effectively reduced AD/DD incidence. **Trial Registration:** The study was preregistered in the German Clinical Trial Registration (DRKS00011099; https://drks.de/search/de/trial/DRKS00011099).

## Introduction

Anxiety and depressive disorders are prevalent mental health conditions that impose substantial personal and economic burden on individuals and society^1^. Despite effective treatments, their prevalence remains high, prompting increased attention towards preventive measures^2^. Leveraging the internet to deliver preventative programs is gaining momentum due to its broad reach, easy access, and potential scalability^3^. Internet- and mobile-based interventions (IMIs) can be offered with varying degrees of human guidance, but automated guidance could present a promising compromise by providing both support and the potential for resource-efficient and widespread scale-up^4,5^. IMIs have been found to effectively reduce subthreshold symptomatology of either anxiety or depressive disorders with small effects^6,7^. However, since mere symptom reduction does not necessarily indicate effective prevention, assessing the reduction in disorder incidence is necessary.

Despite their high comorbidity and substantial psychopathological and pathogenetic overlap, most prevention programs address anxiety and depressive disorders separately. This approach reduces the likelihood of the widespread implementation of concepts for both disorders and limits their potential public health impact. Moreover, most programs use standardized formats, leaving little room to address comorbidities or individual distinctive features. Addressing this comorbidity might necessitate transdiagnostic and tailored interventions^8^, targeting shared mechanisms like avoidance^9^, and customizing module based on participants’ characteristics and preferences^10^.

This indicated prevention study aimed to evaluate the efficacy of a transdiagnostic and self-tailored IMI designed to address the high comorbidity of anxiety and depressive disorders. The study compared the IMI with either individualized (IG-IMI) or automated guidance (IGI-AG) to a waitlist control group (WLC), aiming to investigate the reduction in the onset of full syndrome anxiety and depressive disorders over a 12-month follow-up period. We hypothesize that (1) both the individually guided and the automatically guided IMI are more effective than the WLC, and (2) the individually guided IMI is more effective than the automatically guided IMI.

## Results

### Participant characteristics

**Fig. 1:**
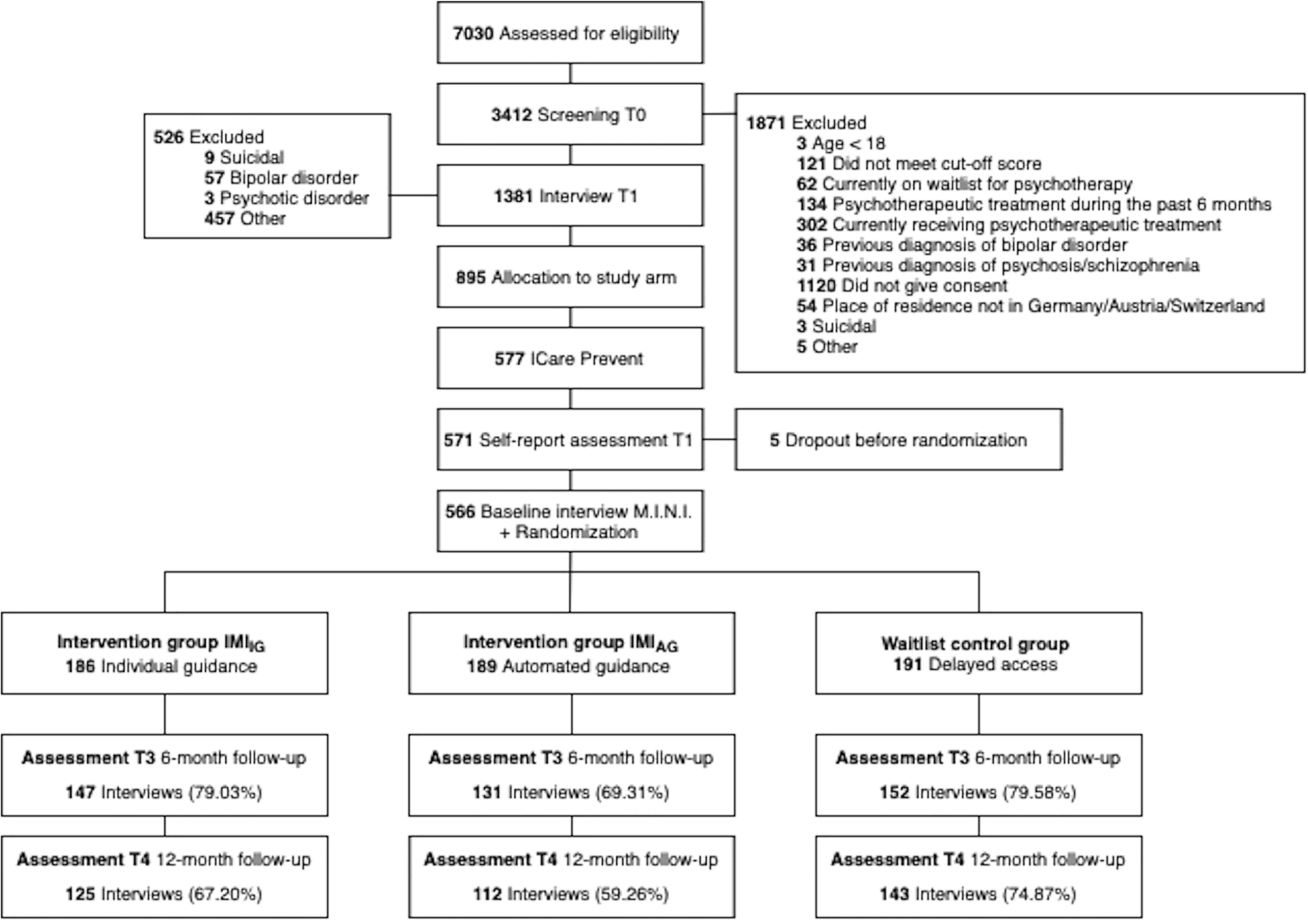
Assessment, randomization, and follow-ups of study participants. Adapted from ref. ^23^. Interviews conducted via the Mini International Neuropsychiatric Interview Version 6.0 for DSM-IV criteria. The figure was created using the diagram tool draw.io.

Figure 1 shows the flow of participants through the study. Between February 7th, 2017, and June 14th, 2019, a total of 566 participants were enrolled in the study (IG-IMI *n* = 186, AG-IMI *n* = 189, WLC *n* = 191) Overall, all participants (n = 566/566, 100%) completed the MINI interview at baseline, 75.97% (*n* = 430/566) at 6-month follow-up, and 67.14% (*n* = 380/566) at 12-month follow-up. When no event was detected, the interview date was used as censoring time point. Patterns of last reported interview dates differed significantly between study arms (*p* = 0.0057 by χ2 test). In the WLC, a larger proportion of participants provided 12-month interview data (74.9%, 143/191) compared to the IG-IMI with 67.2% (*n* = 125/186) and the AG-IMI with 59.3% (112/189). The chance to provide 12-month interview data was independent of baseline symptom severity with respect to HAMA (*p* = 0.8262), QIDS (*p* = 0.8395), GAD-7 (*p* = 0.3343), gender (*p* = 0.3433), and age (*p* = 0.5356). We detected a negative association with CES-D (*p* = 0.0224) and a positive association with intervention adherence in both intervention groups (*p* < 0.0001). Characteristics of participants at study start are presented in Table 1. The average participant was a female, of Caucasian ethnicity, aged 40, and holding a university degree. On average, participants completed 6.84 sessions (SD = 2.02) in the IG-IMI and 6.19 sessions (SD = 2.40) in AG-IMI. Out of 566 participants, 463 (81.8%) presented with both subclinical anxiety and depressive symptoms. Seventy-five participants (13.3%) had isolated anxiety symptoms, and 28 participants (5.0%) had isolated depressive symptoms.

**Table 1:** Baseline characteristics of participants experiencing subthreshold anxiety or depressive symptoms according to study group

Sociodemographic data	IG-IMI (*n* = 186)		AG-IMI (*n* = 189)		WLC (*n* = 191)		Total (*N* = 566)	
Age, mean (SD)	40.61	(14.33)	38.67	(13.68)	41.13	(13.99)	40.14	(14.02)
range	(19-75)		(19-81)		(18-77)		(18-81)	
Female, *n* (%)	140	(75.27)	134	(70.90)	136	(71.20)	410	(72.44)
Germany as country of residence, *n* (%)	152	(81.72)	161	(85.19)	165	(86.39)	478	(84.45)
Migration background, *n* (%)	25	(13.44)	27	(14.29)	22	(11.52)	74	(13.07)
Caucasian, *n* (%)	157	(84.41)	163	(86.24)	164	(85.86)	484	(85.51)
University degree, *n* (%)	112	(60.22)	117	(61.90)	118	(61.78)	347	(61.31)
Single, *n* (%)	72	(38.71)	71	(37.57)	81	(42.41)	224	(39.58)
Place of residence (<20.000 residents)	60	(32.26)	61	(32.28)	54	(28.27)	175	(30.92)
**Mental health treatment experience**								
Prior diagnosis of mental disorder, *n* (%)	62	(33.33)	71	(37.57)	64	(33.51)	197	(34.81)
Depressive disorder, *n* (%)	49	(26.34)	50	(26.46)	47	(24.61)	146	(25.80)
Anxiety disorder/Posttraumatic stress disorder/obsessive compulsive disorder, *n* (%)	27	(14.52)	30	(15.87)	26	(13.61)	83	(14.66)
Prior psychotherapy experience, *n* (%)	93	(50.00)	85	(44.97)	91	(47.64)	269	(47.53)
Prior experience with health trainings, *n* (%)	38	(20.43)	32	(16.93)	51	(26.70)	121	(21.38)

Adapted from^23^.

*AG-IMI* automated guided intervention group, *IG-IMI* individually guided intervention group, *n* number, *SD* standard deviation, *WLC* waitlist control group.

### Efficacy of the intervention

#### Primary analysis

**Fig. 2:**
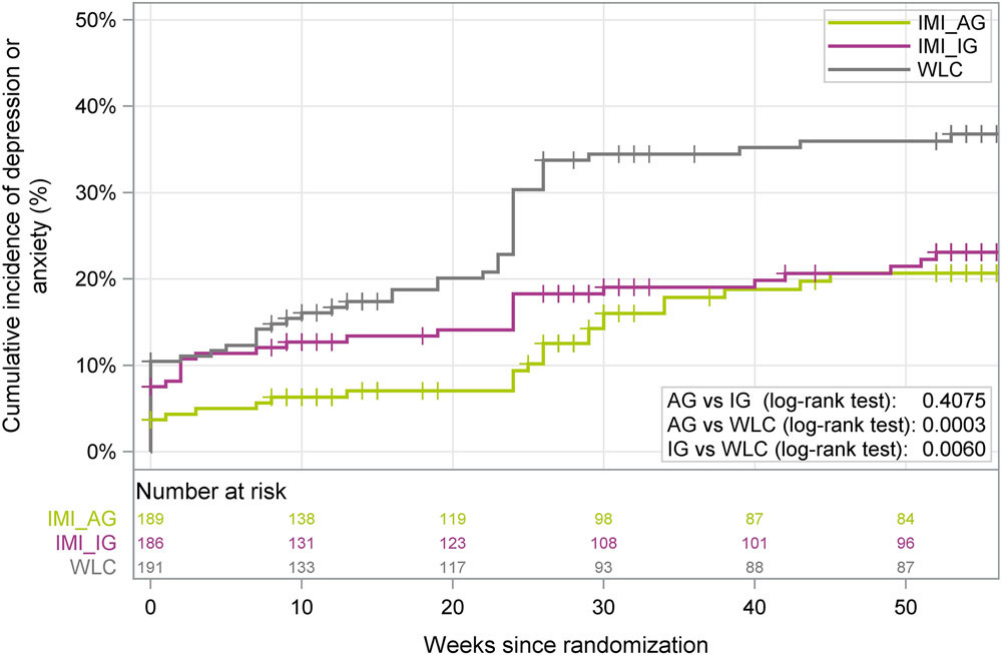
Cumulative incidence function (inverse Kaplan–Meier estimate of time to onset of an anxiety or depressive disorder by study group). The primary endpoint was time to onset in weeks of an anxiety or depressive disorder in the individual or automated guided study condition relative to the control group measured with the Mini International Neuropsychiatric Interview Version 6.0 for DSM-IV criteria^26^. Follow-Up was truncated at 52 weeks. The log-rank test was utilized to examine the hypothesized differences between the groups as per the study design. WLC=Waitlist control group, IG-IMI individually guided group, AG-IMI automated guided group. The figure was created using the statistical software SAS.

AD/DD was experienced by 19.4% participants (*n* = 36/186) in the IG-IMI (AD: *n* = 14/186, 7.5%, DD: *n* = 27/186, 14.5%) and 15.1% participants (*n* = 28/189) in the AG-IMI (AD: *n* = 9/189, 4.8%, DD: *n* = 25/189, 13.2%) in contrast to 30.9% participants (*n* = 59/191) in the WLC (AD: *n* = 29/191, 15.2%, DD: *n* = 43/191, 22.5%). The mean time to onset of AD/DD within the 12-month trial period was 43.53 weeks (95%-CI: 40.81–46.25) in the IG-IMI, 40.12 weeks (95%-CI: 38.27–41.97) in the AG-IMI, and 39.07 weeks (95%-CI: 36.03–42.11) in WLC. Figure 2 illustrates the inverse KM estimates as cumulative incidence plots for both intervention groups, respectively, and the WLC over the 12-month study period.

In IG-IMI, the 12-month cumulative incidence of AD/DD was 23.1% (95%-CI: 17.1–30.7%) in comparison to 36.0% (95%-CI: 29.0–44.0%) in WLC (log-rank test: *adj. p* ≤ 0.01; hazard ratio [HR] = 0.59, 95-CI: 0.39–0.89). In AG-IMI, the 12-month cumulative incidence of AD/DD was 20.7% (95%-CI: 14.6–28.8%) in comparison to WLC (log-rank test: *adj. p* ≤ 0.001; HR = 0.47, 95%-CI: 0.30–0.73) (Supplementary Tables 4 and 5 in Appendix). Given the adjusted local significance levels, both primary null hypotheses could be rejected and an effect of IG-IMI and AG-IMI versus WLC, respectively, could be shown. At 12-month follow-up, the NNT to prevent one new case of AD/DD was 7.76 (95%-CI: 4.99–13.66) for IG-IMI versus WLC and 5.79 (95%-CI: 4.24–12.15) for AG-IMI versus WLC.

For sensitivity analyses on AD/DD incidence rates, the Cox model when controlling for baseline scores revealed a HR of 0.54 (95%-CI: 0.35–0.81) in the IG-IMI and a HR of 0.45 (95%-CI: 0.28–0.70) in the AG-IMI in comparison to WLC (type 3 test, *p* = 0.0004, Supplementary Table 1 in Appendix). The test for proportionality within the randomized groups yielded no substantive evidence contradicting this underlying assumption (*p* = 0.244). Participants with prior reported AD/DD episodes had a similar 12-month cumulative incidence of AD/DD (IG-IMI: 22.6%, AG-IMI: 18.9%, WLC: 35.0%) compared to the primary prevention sub-cohort (IG-IMI: 23.4%, AG-IMI: 21.5%, WLC: 36.4%). No statistically significant differences were observed between participants who had previously experienced AD/DD and those who had not, within each study arm (all *ps* > 0.05). Exploratory analyses of differences in anxiety and depressive disorders as secondary outcomes, comparisons between IG-IMI and AG-IMI for the composite AD/DD outcome, and study and intervention completer analyses are presented in Supplementary Results 1–4 and Supplementary Fig. 1 in Appendix.

## Discussion

To our knowledge, this is one of the first studies to evaluate the efficacy of an indicated prevention program for AD/DD onset in adults. As hypothesized, the results showed that the IMI significantly reduced or delayed combined AD/DD onset in both the individual guidance group (hazard ratio = 0.59, indicating a 41% relative risk reduction) and the automated guidance group (hazard ratio = 0.47, indicating a 53% relative risk reduction) compared to WLC. Exploratory analyses revealed effects on cumulative incidence of anxiety (HR = 0.48 IG-IMI, HR = 0.32 AG-IMI) and depressive disorders (HR = 0.61 AG-IMI), though for depression, IG-IMI reached only borderline significance (*p* = 0.05, HR = 0.48). Findings were independent of first vs. recurrent disorder onset.

Only a limited number of studies have evaluated the impact of indicated prevention programs on the onset of depression and anxiety disorders, all of which focused on older adults to prevent late-life anxiety and depression. Consistent with our findings, Van’t Veer-Tazelaar et al. (2009) demonstrated that a stepped-care program, including watchful waiting, CBT-based bibliotherapy, problem-solving treatment, and pharmacotherapy referral, reduced the 12-month incidence of cumulative anxiety and depression in adults ≥75 years by 49% (from 0.24 to 0.12)^11^. Similarly, a stepped-care program for adults aged ≥50 years with impaired vision and subthreshold anxiety and/or depressive symptoms reduced the risk of developing anxiety or depressive disorders by 43% (HR 0.57)^12^. Two other trials found no effect of a stepped-care program in reducing the combined incidence of anxiety and depressive disorders in patients with subthreshold symptoms^13,14^.

Compared to prior depression prevention trials reporting an average 19 to 21% reduction in incidence^15,16^, our study’s HR showed greater effectiveness. This difference may stem from variations in intervention content, format, or participant characteristics, such as baseline vulnerabilities. Indicated prevention of anxiety disorders has been less studied in terms of disorder onset, with no additional trials beyond the mentioned stepped-care programs identified in the latest meta-analysis^17^. The present results exceeded those reported for unguided IMIs in a meta-analysis, which found an overall Risk Ratio of 0.67 for depression and no significant effect on anxiety incidence, highlighting the potential importance of guidance and the application of automated guidance formats in unguided IMIs^18^. The results for the individually guided condition align with a trial by our group on an IMI for the indicated prevention of depression (HR = 0.59)^19^. This suggests that simultaneously targeting depression and various anxiety disorders is feasible without compromising effectiveness, thereby enhancing the reach and public health impact of such interventions.

Comparable effects of individualized and automatic guidance, contrary to our hypothesis, align with recent meta-analytic findings showing no differences between guided and unguided IMIs for subthreshold depressive symptoms^20^. The results emerged despite lower adherence in the automated condition, suggesting that even a lower treatment dose may be sufficient. Standardized messages may have been adequate, given the intervention’s intensive content, rich psychoeducation, and the fact that nearly half of the sample had prior psychotherapy experience. The main role of guidance may lie in fostering motivation, which could be addressed through automated reminders. Alternatively, the limited added benefit of individual guidance may reflect a mismatch between participants’ expectations and the semi-standardized support provided by the eCoach, who did not offer psychotherapy.

This trial also adds to the literature by showing that this preventive approach is effective for individuals with and without prior episodes of anxiety or depression, addressing both first onset and recurrence -limitations of many earlier trials^19^.

There are limitations to this study. First, while it presented data from German-speaking countries, the trial was powered as a multinational study conducted across four European countries^21^. Deviations from the initial study protocol at excluded sites required separate analyses^22^. Hypothetically, an available sample size of 185 participants per group would have provided sufficient power (>80%) to detect a hazard ratio of 0.57 or lower between an intervention arm and the control condition. Two pre-planned subgroup analyses for comorbidities, specifically alcohol abuse and specific anxiety disorders, could not be conducted due to insufficient cases in each category. Second, this study employed an extensive screening procedure, which might have resulted in the inclusion of participants particularly motivated for behavior change. Consequently, findings from this study may not generalize to routine preventive care settings without such intensive screening. Future research is needed to evaluate effectiveness under real-world conditions. Third, prior diagnoses at baseline were established by self-report, asking participants whether they had been previously diagnosed with depression or anxiety disorders. The latter category included posttraumatic stress disorder and obsessive-compulsive disorder, potentially leading to an overestimation of pre-existing anxiety disorders. Fourth, while all participants had access to routine health care services, data on service usage was not fully reported during the assessment. Fifth, nearly half of the participants (47.53%) had prior psychotherapy experience, likely due to the study’s focus on preventing both first onset and recurrence of anxiety and depression, and the high proportion with prior mental disorders. This may limit generalizability, as these participants could differ from the general population in help-seeking behavior, symptom awareness, and fewer stigma-related barriers, potentially affecting how the intervention translates to those without prior treatment. Notably, results did not differ by anxiety or depression history. Sixth, other mental health comorbidities (e.g., somatoform, personality, or substance use disorders) were not assessed at baseline or considered in the randomization, which may have influenced both disorder onset and intervention response. Seventh, the interrater reliability (*κ* = 0.77) was based on a small subsample at baseline (*n* = 55) and may not fully reflect reliability at follow-ups, which represents a potential limitation. Eighth, 12-month follow-up completion differed between groups, potentially reflecting varying motivation. Intervention participants may have felt less compelled to continue after accessing the program, while WLC participants remained engaged to gain later access. Higher adherence with individual versus automated guidance may reflect greater emotional engagement and accountability through personalized feedback. Nonetheless, both formats were effective, suggesting even lower-intensity interventions may offer preventive benefits. Missing outcome data were handled using appropriate statistical methods to reduce potential bias.”

Future studies should aim to identify active intervention components and explore strategies to optimize efficacy. This includes tailoring interventions to specific individual vulnerabilities and leveraging AI-based approaches to personalize approaches. In particular, large language models could enhance personalization by analyzing user input in real time to deliver adaptive feedback, recommend modules based on symptom profiles, or simulate coaching interactions through conversational agents. Furthermore, tracking elective session selection patterns could help determine whether specific module choices influence outcomes. In this sample, 64.6% of participants (*n* = 366/566) expressed a preference for receiving feedback, while 31.45% (*n* = 178/566) had no guidance preference. Future research should examine whether receiving one’s preferred type of feedback is associated with improved outcomes. Additionally, evaluating the cost-effectiveness of individual versus automated guidance formats in prevention is warranted, as both demonstrated comparable clinical outcomes but may differ substantially in resource demands.

Among individuals with subthreshold anxiety or depressive symptoms, the use of an IMI with either individual or automated guidance compared to WLC reduced the incidence of a full clinical anxiety or depressive disorder over 12 months. Thus, providing easily accessible evidence-based preventive interventions through the internet could potentially serve as a strategy to reach individuals in the early stages, aiding in preventing the progression from subthreshold anxiety or depressive symptoms to a clinical disorder, or in preventing relapses in cases of recurrent disorders.

## Methods

### Trial design and participants

A three-group randomized controlled trial (RCT) assessed the efficacy of an IMI in an individually and automated guided version compared to a WLC for anxiety or depressive disorder onset. The study was preregistered (German Clinical Trial Registration DRKS00011099) and a study protocol has been published^21^, along with secondary outcomes of the study, i.e., symptom severity outcomes^23^. For this primary outcome incidence analysis, data were collected at baseline, 6- and 12-month follow-up at the German-speaking trial sites, including participants from Germany, Switzerland, and Austria. Approval was obtained from ethics committees in both Germany and Switzerland (medical ethics committee University of Erlangen-Nürnberg, Germany, reference No. 144_16 B; ethics commission of the Canton of Bern, Switzerland, reference No. 2016-01389). Deviating from the study registration, data from the trial sites in Spain and the Netherlands were excluded from the analyses due to protocol deviations caused by local regulatory requirements, which led to incomparability of samples and procedures. This resulted in a final sample size of *N* = 566 instead of the originally planned *N* = 957 ^22^.

Participants were recruited through various online and offline channels including the study website, Google AdWords, online magazines, and social media platforms like Facebook and Instagram, as well as flyers and health insurance company mail campaigns. To determine eligibility, potential participants completed an online screening process ensuring that they: (1) were ≥18 years, (2) experienced subclinical anxiety (Generalized Anxiety Disorder Scale-7 [GAD-7] score ≥5^24^) and/or depressive (Centre for Epidemiological Studies Depression Scale [CES-D] score ≥16^25^ symptoms)), (3) had no current or past diagnosis of psychosis, bipolar disorder, or dissociative symptoms, (4) did not exhibit heightened suicidal risk, (5) had internet access and a valid email address, (6) possessed adequate German reading and writing skills, (7) provided informed consent, and (8) were not currently undergoing psychotherapy or were on a therapy waitlist, and had not undergone psychotherapy for AD/DD in the past six months. Potential eligible participants underwent the Mini International Neuropsychiatric Interview Version 6.0 (MINI^26^) for DSM-IV criteria via telephone, and individuals fulfilling either of the following criteria were excluded: (1) met full diagnostic criteria for an anxiety disorder within the past six months, or (2) met full diagnostic criteria for major depression within the past six months and reported a core symptom of major depression (such as dysphoria or anhedonia) within the past three weeks.

### Randomization and masking

Individual randomization occurred at 1:1:1 ratio (IG-IMI, AG-IMI, WLC) based on the type of subclinical symptom presence (subclinical anxiety, subclinical depression, comorbid subclinical anxiety disorder and subclinical depressive disorder) within three strata. The study statistician independently generated randomization lists using automated block-randomization with concealed block lengths. Participants were randomized online by study personnel, ensuring allocation concealment of future randomizations for study personnel, clinical interviewers, eCoaches, and participants. After randomization, IG-IMI participants were informed that they would receive feedback from an eCoach, while AG-IMI participants would receive no additional information. Clinical interviewers remained blind to participants’ randomization status. If inadvertent unblinding occurred during outcome interviews, the potentially unblinded clinical interviewer was replaced by a different interviewer for subsequent follow-up telephone assessments. At T2, 18 out of 396 interviews (4%) and at T3, 5 out of 275 interviews (1%) were potentially unblinded, requiring a mandatory change of interviewer for the follow-up. At T4, 5 out of 209 interviews (1%) were also potentially unblinded.

### Intervention

Both intervention groups received the same 7-session IMI targeting anxiety and depressive symptoms, which included transdiagnostic elements like behavioral activation, cognitive restructuring, psychoeducation, problem-solving, exposure, and relapse prevention. An additional booster session was provided four weeks after the seventh session. There were eight elective sessions covering topics such as rumination, acceptance, relaxation, alcohol, self-worth, perfectionism, appreciation, gratitude, and sleep. The intervention was accessible via a secure, browser-based eHealth platform compatible with both computers and mobile devices. Sessions were individually tailored, allowing participants to select components that suited them best (e.g., problem-solving, exposure, or both) after receiving psychoeducation on anxiety and depressive symptoms, and optional motivational messages were accessible via smartphone. The content included text, audio, and video formats, along with a smartphone diary feature. A more detailed description of the intervention is available in the study protocol^21^.

#### Individual guidance

In the IG-IMI, participants received personalized feedback for each session, tailored to their progress and input, following the guidance manual for the study. This feedback was provided by a dedicated eCoach through the platform’s messaging function. These eCoaches had psychology degrees and received supervision as needed. On average, eCoaches spent 90 min per participants (around 13 min per session) on guidance in total. Adherence monitoring included personalized reminders via email and text if a session remained incomplete after seven days.

#### Automated guidance

In the AG-IMI, participants received standardized automated feedback on completed sessions via the eHealth platform, without personal adaptations by an eCoach. The aim of this feedback was to enhance engagement. Study administrators sent adherence reminders for technical reasons, without incorporating any personalized communication.

### Outcomes

Primary outcome was time to onset of any AD/DD in IG-IMI and AG-IMI compared to WLC, respectively, over a 12-month follow-up period. We used DSM-IV criteria assessed with MINI 6.0^26^ at baseline, 6-, and 12 months. A subsample of the baseline interviews (k = 55) was recorded and rated for interrater reliability resulting in a κ coefficient of 0.77 for AD/DD diagnosis agreement, indicating substantial agreement. To mitigate recall bias, we determined AD/DD onset time (in weeks since randomization) using the Life Chart Method^27^. This method links personal milestones to the calendar, helping to pinpoint symptom timelines and life events. When exact dates were unclear, we selected the nearest week (or month) midpoint for accuracy.

### Statistical analyses

The study tested two primary statistical hypotheses concerning the reduction in the incidence of AD/DD (1) IG-IMI vs. WLC and (2) AG-IMI vs. WLC. Exploratory evaluations of differences in either AD or DD as secondary outcomes were also conducted (Supplementary Results 1 in Appendix), as well as an exploratory comparison between IG-IMI and AG-IMI (Supplementary Results 2 and Supplementary Fig. 1 in Appendix). The initial power analysis assumed a mean 12-month incidence rate of AD/DD of 15% in the intervention conditions and of 25% in the WLC, representing a 40% reduction in relative cumulative risk^15,28^.

Reporting followed the CONSORT statement^29^. Crude event rates are reported as absolute and relative frequencies. Data analysis was conducted on an intention-to-treat basis, including all randomly assigned participants. For the time-to-event analyses, all observation times were included in the Kaplan–Meier estimates, regardless of whether an AD/DD event occurred. Missing data regarding the event of interest were handled through right-censoring: participants lost to follow-up or who completed the follow-up without experiencing an AD/DD contributed observation time up to their last available time point. Censoring was accounted for via the at-risk set in each analysis.

To assess differences in time to onset of AD/DD (in weeks) between each intervention group and the WLC, survival curves were estimated using Kaplan–Meier methodology. Inverse Kaplan–Meier (KM) estimates (1-KM) as cumulative incidence plots, along with the number-at-risk, were reported, and cumulative incidence estimates in percent (95% confidence interval) at 12-M-FU for all study conditions were presented. Cumulative incidence estimates between IG-IMI and AG-IMI, respectively, and WLC were compared by two-sided log rank tests with a Bonferroni-Holm adjustment for multiple testing.

The number needed-to-treat (NNT) for IG-IMI and AG-IMI to prevent one additional case of AD/DD compared to WLC was calculated^30^. Additionally, a per-protocol analysis examined differences in time to onset of AD/DD in study and intervention completers (Supplementary Results 3 and 4 in Appendix). Sensitivity analyses used a multivariable Cox proportional hazard regression model, adjusting for age, gender, type of subclinical symptoms (subclinical AD, DD, or both), baseline symptom severity (GAD-7, CES-D, HAM-A, QIDS), history of mental health disorder, and previous treatment experience, testing the proportional hazards assumption based on the scaled Schoenfeld residuals test^31^ (Supplementary Tables 1–3 in Appendix).

## Supplementary information

Supplementary Information

CONSORT_2025_editable_checklist.

## Data Availability

Deidentified participant data will be available upon request from the authors, including a data dictionary.

## References

[1] Vos, T. et al. Global burden of 369 diseases and injuries in 204 countries and territories, 1990–2019: a systematic analysis for the Global Burden of Disease Study 2019. *Lancet* **396**, 1204–1222 (2020). 33069326 10.1016/S0140-6736(20)30925-9 PMC7567026

[2] Cuijpers, P. et al. Psychological interventions to prevent the onset of depressive disorders: a meta-analysis of randomized controlled trials. *Clin. Psychol. Rev.* **83**, 101955 (2021). 10.1016/j.cpr.2020.101955 33333441

[3] Ebert, D. D., Cuijpers, P., Muñoz, R. F. & Baumeister, H. Prevention of mental health disorders using internet and mobile-based interventions: a narrative review and recommendations for future research. *Front. Psychiatry* **8**, 116 (2017). 28848454 10.3389/fpsyt.2017.00116 PMC5554359

[4] Shim, M., Mahaffey, B., Bleidistel, M. & Gonzalez, A. A scoping review of human-support factors in the context of Internet-based psychological interventions (IPIs) for depression and anxiety disorders. *Clin. Psychol. Rev.* **57**, 129–140 (2017). 28934623 10.1016/j.cpr.2017.09.003

[5] Musiat, P., Johnson, C., Atkinson, M., Wilksch, S. & Wade, T. Impact of guidance on intervention adherence in computerised interventions for mental health problems: a meta-analysis. *Psychol. Med.* **52**, 229–240 (2022). 34802474 10.1017/S0033291721004621

[6] Sander, L., Rausch, L. & Baumeister, H. Effectiveness of internet-based interventions for the prevention of mental disorders: a systematic review and meta-analysis. *JMIR Ment. Health* **3**, e38 (2016). 27535468 10.2196/mental.6061 PMC5007382

[7] Deady, M. et al. eHealth interventions for the prevention of depression and anxiety in the general population: a systematic review and meta-analysis. *BMC Psychiatry* **17**, 1–14 (2017). 28851342 10.1186/s12888-017-1473-1 PMC5576307

[8] Andrews, G. & Williams, A. D. Internet psychotherapy and the future of personalized treatment. *Depress Anxiety* **31**, 912–915 (2014). 25407580 10.1002/da.22302

[9] Norton, P. J. & Paulus, D. J. Toward a unified treatment for emotional disorders: update on the science and practice. *Behav. Ther.* **47**, 854–868 (2015). 27993337 10.1016/j.beth.2015.07.002

[10] Carlbring, P. et al. Individually-tailored, internet-based treatment for anxiety disorders: a randomized controlled trial. *Behav. Res. Ther.* **49**, 18–24 (2011). 21047620 10.1016/j.brat.2010.10.002

[11] Van’t Veer-Tazelaar, P. J. et al. *Stepped-Care Prevention of Anxiety and Depression in Late Life A Randomized Controlled Trial*. *Arch. Gen. Psychiatry* **66**, 297–304 (2009). 10.1001/archgenpsychiatry.2008.555 19255379

[12] Van Der Aa, H. P. A. et al. Stepped care for depression and anxiety in visually impaired older adults: multicentre randomised controlled trial. *BMJ* **351**, 351:h6127 (2015). 26597263 10.1136/bmj.h6127 PMC4655616

[13] Zhang, D. X. et al. Prevention of anxiety and depression in Chinese: a randomized clinical trial testing the effectiveness of a stepped care program in primary care. *J. Affect Disord.* **169**, 212–220 (2014). 25216464 10.1016/j.jad.2014.08.015

[14] Dozeman, E. et al. Contradictory effects for prevention of depression and anxiety in residents in homes for the elderly: a pragmatic randomized controlled trial. *Int. Psychogeriatr.* **24**, 1242–1251 (2012). 22436082 10.1017/S1041610212000178

[15] Van Zoonen, K. et al. Preventing the onset of major depressive disorder: a meta-analytic review of psychological interventions. *Int. J. Epidemiol.* **43**, 318–329 (2014). 24760873 10.1093/ije/dyt175 PMC4023317

[16] Cuijpers, P. et al. Psychological interventions to prevent the onset of depressive disorders: a meta-analysis of randomized controlled trials. *Clin. Psychol. Rev.* **83**, 1–13 (2021). 10.1016/j.cpr.2020.101955 33333441

[17] Moreno-Peral, P. et al. Effectiveness of psychological and/or educational interventions in the prevention of anxiety: a systematic review, meta-analysis, and meta-regression. *JAMA Psychiatry* **74**, 1021–1029 (2017). 28877316 10.1001/jamapsychiatry.2017.2509 PMC5710546

[18] Edge, D., Watkins, E. R., Limond, J. & Mugadza, J. The efficacy of self-guided internet and mobile-based interventions for preventing anxiety and depression – a systematic review and meta-analysis. *Behav. Res. Ther.* **164**, 104292 (2023). 10.1016/j.brat.2023.104292 37003138

[19] Buntrock, C. et al. Effect of a web-based guided self-help intervention for prevention of major depression in adults with subthreshold depression a randomized clinical trial. *J. Am. Med. Assoc.* **315**, 1854–1863 (2016). 10.1001/jama.2016.4326 27139058

[20] Karyotaki, E. et al. Internet-based cognitive behavioral therapy for depression: a systematic review and individual patient data network meta-analysis. *JAMA Psychiatry* **78**, 361–371 (2021). 33471111 10.1001/jamapsychiatry.2020.4364 PMC8027916

[21] Weisel, K. K. et al. Efficacy and cost-effectiveness of guided and unguided internet- and mobile-based indicated transdiagnostic prevention of depression and anxiety (ICare Prevent): A three-armed randomized controlled trial in four European countries. *Internet Interv.* **16**, 52–64 (2019). 30775265 10.1016/j.invent.2018.04.002 PMC6364519

[22] Bolinski, F. et al. Challenges in recruiting university students for web-based indicated prevention of depression and anxiety: results from a randomized controlled trial (ICare Prevent). *J. Med. Internet Res.* **24**, e40892 (2022). 10.2196/40892 PMC9798269 36515986

[23] Zarski, A.-C. et al. Efficacy of an internet-and mobile-based intervention for subclinical anxiety and depression (ICare Prevent) with two guidance formats: results from a three-armed randomized controlled trial. *Psychother. Psychosomatics* **93**, 155–168 (2024). 10.1159/000536149 PMC11151970 38688243

[24] Spitzer, R. L., Kroenke, K., Williams, J. B. & Löwe, B. A brief measure for assessing generalized anxiety disorder. *Arch. Intern. Med.* **166**, 1092–1097 (2006). 16717171 10.1001/archinte.166.10.1092

[25] Radloff, L. S. The CES-D scale: a self-report depression scale for research in the general population. *Appl. Psychol. Meas.* **1**, 385–401 (1977).

[26] Sheehan, D., Janavs, J., Harnett-Sheehan, K., Sheehan, M. & Gray, C. Mini International Neuropsychiatric Interview. German Translation Version 6.0.0. (2010). Available from: Mapi Research Trust, Lyon, France.

[27] Lyketsos, C., Nestadt, G., Cwi, J. & Heithoff, K. The life chart interview: a standardized method to describe the course of psychopathology. *Int. J. Methods Psychiatr. Res.* (1994).4(3):143–155

[28] Van’t Veer-Tazelaar, P. J. et al. Stepped-care prevention of anxiety and depression in late life. *Arch. Gen. Psychiatry* **66**, 297–304 (2009). 19255379 10.1001/archgenpsychiatry.2008.555

[29] Schulz, K. F., Altman, D. & Moher, D. Statement: uUpdated guidelines for reporting parallel group randomised trials. *J. Pharm. Pharmacother.* **1**, 100 (2010). CONSORT 2010. 10.4103/0976-500X.72352 PMC3043330 21350618

[30] Altman, D. G. & Andersen, P. K. Calculating the number needed to treat for trials where the outcome is time to an event. *Br. Med. J.* **319**, 1492–1495 (1999). 10582940 10.1136/bmj.319.7223.1492 PMC1117211

[31] Grambsch, P. M. & Therneau, T. M. Proportional hazards tests and diagnostics based on weighted residuals. *Biometrika* **81**, 515–526 (1994).

