# Supplementary material for: Internet-based indicated prevention of anxiety and depression disorder onset three-arm randomized clinical trial
Source: NPJ Digit Med. 2025 Oct 1;8:589. doi: 10.1038/s41746-025-01990-8 (PMC12489038; doi:10.1038/s41746-025-01990-8)
Supplement: Supplementary file 1 — Supplementary Information [file 41746_2025_1990_MOESM1_ESM.pdf]

Appendix in Supplementary Online Content

This supplementary material has been provided by the authors to give readers additional information about their work.

Supplementary Results 1. Exploratory evaluations of differences in either anxiety or depressive disorders as secondary outcomes.

In the individually guided condition (IG-IMI), 7.5% participants (n=14/186) experienced anxiety disorders (AD) and 14.5% (n=27/186) experienced depressive disorders (DD), while in the automated guided condition (AG-IMI), 4.8% participants (n=9/189) experienced AD and 13.2% participants (25/189) DD in contrast to 15.2% participants (29/191) with AD and 22.5% (43/191) with DD in the waitlist control condition (WLC).

The cumulative incidence of AD for WLC group was 17.4% (95%-CI: 12.4% – 24.1%) at 12-month. The 12-month cumulative incidence of AD was 9.1% (95%-CI: 5.5% – 15.0%) for IG-IMI (log-rank test: *p*<.01, HR=0.48, 95%-CI: 0.25-0.90). The 12-month cumulative incidence of AD was 6.2% (95%-CI: 3.3% – 11.7%) for AG-IMI (log-rank test: *p*<.001, HR=0.32, 95%-CI: 0.15-0.68).

The 12-month cumulative incidence of DD for WLC was 26.5% (95%-CI: 20.3% – 34.2%). The 12-month cumulative incidence of DD was 17.3% (95%-CI: 12.1% – 24.4%) for IG-IMI (log-rank test: *p*=.05, HR=0.48, 95%-CI: 0.25-0.90). The 12-month cumulative incidence of DD was 18.8% (95%-CI: 13.0% – 26.7%) for AG-IMI (log-rank test: *p*=.04, HR=0.61, 95%-CI: 0.38-1.00).

Supplementary Results 2. Exploratory comparison between IG-IMI and AG-IMI in the composite AD/DD outcome.

The 12-month cumulative incidence of AD/DD between IG-IMI und AG-IMI was non-significant (log-rank test: *p*=0.41; HR=0.79, 95-CI: 0.48-1.29).

Supplementary Results 3. Study completer analyses.

The mean time to onset of AD/DD within the 12-month trial period was 42.8 weeks (95%-CI: 39.6-46.1) in the IG-IMI, 38.4 weeks (95%-CI: 38.8-41.0) in the AG-IMI, and 37.3 weeks (95%-CI: 33.9-40.8) in WLC. Note: The mean survival time and its standard error were underestimated because the largest observ ation was censored, and the estimation was restricted to the largest event time. In IG-IMI, the 12-month cumulative incidence of AD/DD was 24.8% (95%-CI: 18.1%-33.3%) in comparison to 39.2% (95%-CI: 31.7%-47.7%) in WLC (log-rank test IG vs WLC: adj. *p*=0.0158; hazard ratio [HR]=0.565, 95-CI: 0.365-0.876). In AG-IMI, the 12-month cumulative incidence of AD/DD was 25.0% (95%-CI: 18.0%-34.1%) in comparison to WLC (log-rank test AG vs WLC: adj. *p*=.0.0139; HR= 0.556, 95-CI: 0.353-0.874).

Supplementary Results 4. Intervention completer analyses.

The mean time to onset of AD/DD within the 12-month trial period was 42.4 weeks (95%-CI: 39.4-45.5) in the IG-IMI, 41.2 weeks (95%-CI: 39.2-43.2) in the AG-IMI, and 39.1 weeks (95%-CI: 36.0-42.1) in WLC. In IG-IMI, the 12-month cumulative incidence of AD/DD was 23.0% (95%-CI: 16.5%-31.4%), significantly lower in comparison to 35.9% (95%-CI: 29.0%-44.0%) in WLC (log-rank test IG vs WLC: adj. *p*=0.0186; hazard ratio [HR]=0.586, 95-CI: 0.376-0.915). In AG-IMI, the 12-month cumulative incidence of AD/DD was 17.7% (95%-CI: 11.3%-26.9%), significant lower in comparison to WLC (log-rank test AG vs WLC: adj. *p*<.001; HR=0.400, 95%-CI: 0.233-0.686).

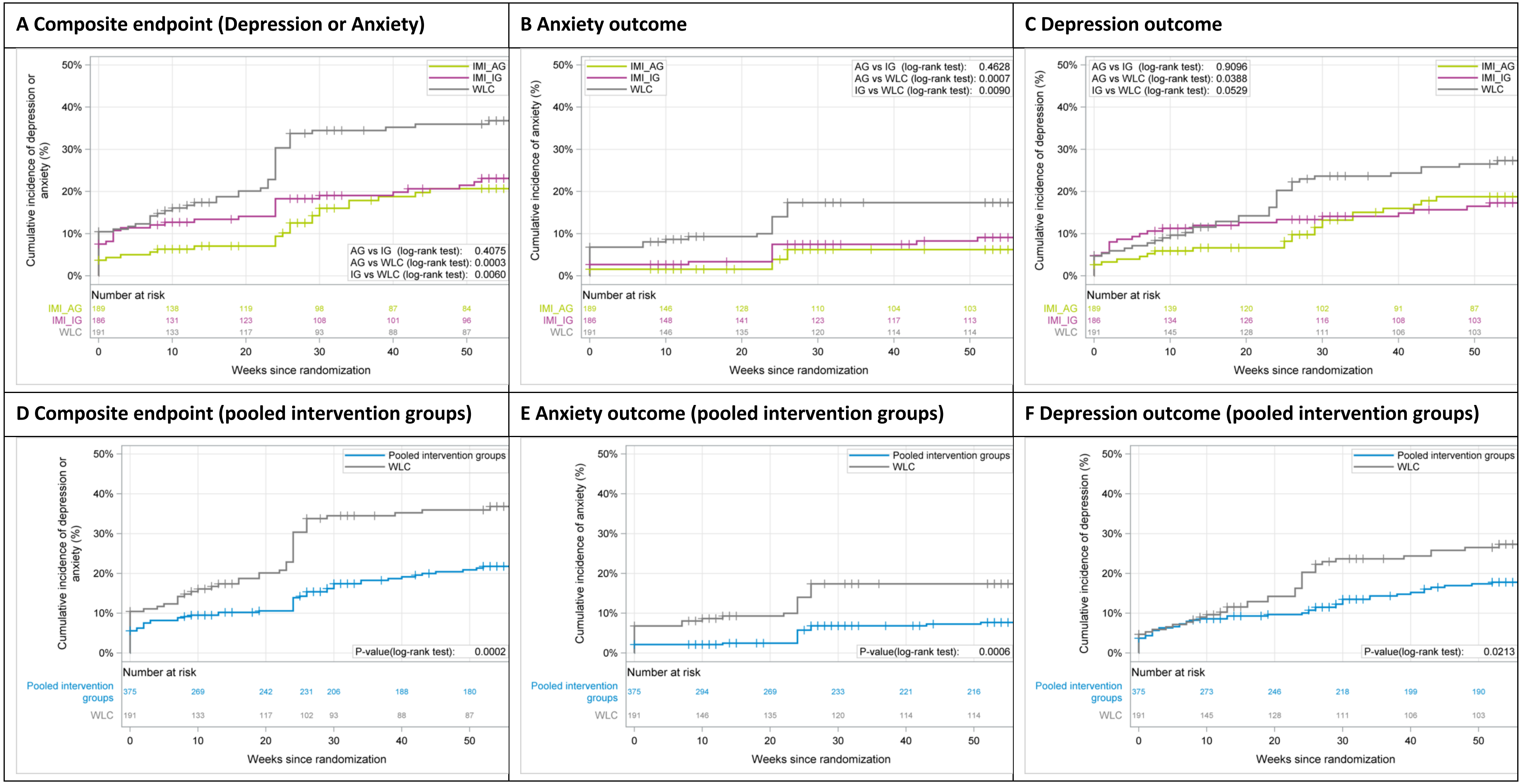

Supplementary Figure 1. Cumulative incidence estimates (1 minus Kaplan-Meier) of the onset of depression or anxiety. All p-values are displayed without adjustment for multiple comparisons.

Supplementary Table 1. Multivariable Cox proportional hazard regression model for AD/DD outcome.

	**Hazard Ratio (95% CI)**	***P***
AG vs. TAU	0.45 (0.28-0.70)	<.001
IG vs. TAU	0.54 (0.35-0.81)	
AG vs. IG	0.84 (0.51-1.37)	
Gender (ref=male)	1.23 (0.81-1.86)	.32
Age (per year)	1.00 (0.98-1.01)	.52
SA vs. SB	0.47 (0.17-1.26)	.16
SA vs. SD	1.24 (0.23-6.71)	
SB vs. SD	2.65 (0.62-11.26)	
GAD-7[1]	0.99 (0.94-1.05)	.82
CES-D[1]	1.05 (1.01 -1.08)	.009
QIDS-C[1]	1.01 (0.95-1.08)	.75
HAMA[1]	1.03 (1.00 -1.06)	.08
History of Mental Disorder[2]	0.83 (0.52-1.31)	.42
Previous treatment experience[2]	0.96 (0.61-1.52)	.87

Abbreviations: [1]per score point, [2]no, reference: no, AD=anxiety disorder, AG=automated guided intervention group, CES-D=Center for Epidemiological Studies Depression Scale, composite=composite anxiety and/or depressive disorder, D=depression disorder, GAD-7=General Anxiety Disorder Measurement, HAM-A=Hamilton Anxiety Rating Scale, IG=individually guided intervention group, *p*=*p*-value, QIDS-C=Quick Inventory of Depressive Symptomatology, SA=subgroup solely subclinical anxiety symptoms at baseline, SB= subgroup both subclinical anxiety and depressive symptoms at baseline, SD= subgroup solely subclinical depressive symptoms at baseline, WCG=waitlist control group

Supplementary Table 2. Multivariable Cox proportional hazard regression model for the DD outcome.

	**Hazard Ratio (95% CI)**	***P***
AG vs. TAU	0.58 (0.35-0.96)	.04
IG vs. TAU	0.59 (0.36-0.96)	
AG vs. IG	0.98 (0.57-1.70)	
Gender (ref=male)	1.29 (0.81-2.04)	.28

Age (per year)	0.99 (0.97-1.01)	.16
SA vs. SB	0.91 (0.29-2.82)	.89
SA vs. SD	1.29 (0.22-7.52)	
SB vs. SD	1.43 (0.33-6.17)	
GAD-7[1]	1.00 (0.94-1.07)	.99
CES-D[1]	1.08 (1.04 -1.12)	<.001
QIDS-C[1]	1.07 (1.00-1.14)	.07
HAM-A[1]	1.00 (0.96 -1.03)	.79
History of Mental Disorder[2]	1.09 (0.65-1.84)	.75
Previous treatment experience[2]	0.94 (0.56-1.58)	.82

Abbreviations: [1]per score point, [2]no, reference: no, AD=anxiety disorder, AG=automated guided intervention group, CES-D=Center for Epidemiological Studies Depression Scale, composite=composite anxiety and/or depressive disorder, D=depression disorder, GAD-7=General Anxiety Disorder Measurement, HAM-A=Hamilton Anxiety Rating Scale, IG=individually guided intervention group, *p*=*p*-value, QIDS-C=Quick Inventory of Depressive Symptomatology, SA=subgroup solely subclinical anxiety symptoms at baseline, SB= subgroup both subclinical anxiety and depressive symptoms at baseline, SD= subgroup solely subclinical depressive symptoms at baseline, WCG=waitlist control group

Supplementary Table 3. Multivariable Cox proportional hazard regression model for the AD outcome.

	**Hazard Ratio (95% CI)**	***P***
AG vs. TAU	0.322 (0.151-0.684)	.003
IG vs. TAU	0.432 (0.226-0.824)	
AG vs. IG	0.745 (0.318-1.742)	
Gender (ref=male)	1.106 (0.579-2.112)	.76
Age (per year)	1.012 (0.991-1.034)	.25
SA vs. SB	0.331 (0.070-1.560)	.38
SA vs. SD	1.29 (0.22-7.52)	
SB vs. SD	1.43 (0.33-6.17)	
GAD-7[1]	1.045 (0.953-1.146)	.35
CES-D[1]	0.999 (0.950 -1.050)	.97
QIDS-C[1]	0.961 (0.872-1.059)	.42

HAM-A[1]	1.069 (1.021 -1.118)	*<.01*
History of Mental Disorder[2]	0.673 (0.338-1.342)	.26
Previous treatment experience[2]	0.813 (0.403-1.642)	.56

Abbreviations: [1]per score point, [2]no, reference: no, AD=anxiety disorder, AG=automated guided intervention group, CES-D=Center for Epidemiological Studies Depression Scale, composite=composite anxiety and/or depressive disorder, D=depression disorder, GAD-7=General Anxiety Disorder Measurement, HAM-A=Hamilton Anxiety Rating Scale, IG=individually guided intervention group, *p*=*p*-value, QIDS-C=Quick Inventory of Depressive Symptomatology, SA=subgroup solely subclinical anxiety symptoms at baseline, SB= subgroup both subclinical anxiety and depressive symptoms at baseline, SD= subgroup solely subclinical depressive symptoms at baseline, WCG=waitlist control group

Supplementary Table 4. Cumulative incidences (1-KM) and 95%-CI at 12 months (52 weeks) in %.

**Treatment Group**	**Composite (AD/DD)**	**AD**	**DD**
Pooled AG/IG	21.8 (17.4-27.1)	7.7 (5.2-11.4)	17.8 (13.8-22.8)
IG	23.1 (17.1-30.7)	9.1 (5.5-15.0)	17.3 (12.1-24.4)
AG	20.7 (14.6-28.8)	6.2 (3.3-11.7)	18.8 (13.0-26.7)
WLC	36.0 (29.0-44.0)	17.4 (12.4-24.1)	26.5 (20.3-34.2)

Abbreviations: AG=automated guided intervention group, AD=anxiety disorder, composite=composite anxiety and/or depressive disorder, DD=depression disorder, IG=individually guided intervention group, KM= Kaplan Meier, WLC=waitlist control condition,

Supplementary Table 5. Univariate Cox proportional hazard regression models.

	**Composite (AD/DD)**		**AD**		**DD**	
	Hazard Ratio (95% CI)	*p*	Hazard Ratio (95% CI)	*p*	Hazard Ratio (95% CI)	*p*
IG vs. WLC	0.59 (0.39-0.89)		0.48 (0.25-0.90)		0.48 (0.25-0.90)	
AG vs. WLC	0.47 (0.30-0.73)	.001	0.32 (0.15-0.68)	.004	0.61 (0.38-1.00)	.08
AG vs. IG	0.79 (0.48-1.29)		0.67 (0.29-1.55)		0.64 (0.40-1.04)	
Pooled vs. WLC	0.53 (0.37-0.75)	<.001	0.40 (0.23-0.69)	.001	0.63 (0.42-0.94)	.02

Abbreviations: AG=automated guided intervention group, AD=anxiety disorder, DD=depression disorder, IG=individually guided intervention group, WLC=waitlist control group
